# High Serum VE-Cadherin and Vinculin Concentrations Are Markers of the Disruption of Vascular Integrity during Type B Acute Aortic Dissection

**DOI:** 10.3390/jcm12144730

**Published:** 2023-07-17

**Authors:** Shiyue Wang, Xin Li, Han Jiang, Jian Zhang

**Affiliations:** Department of Vascular & Thyroid Surgery, The First Hospital of China Medical University, Shenyang 110001, China; 20211016@cmu.edu.cn (S.W.); 2020110147@stu.cmu.edu.cn (X.L.); jianghan@cmu1h.com (H.J.)

**Keywords:** vascular endothelial cadherin, vinculin, acute aortic dissection, endothelial cell, biomarker

## Abstract

Background: In the present study, we measured the serum vascular endothelial cadherin (VEC) and vinculin (Vcn) concentrations in patients with type B acute aortic dissection (TBAD) to evaluate their diagnostic value for this condition. Methods: A total of 100 patients with TBAD and 90 matched controls were included in the study. The serum concentrations of VEC and Vcn were measured using enzyme-linked immunosorbent assays. Results: The serum VEC and Vcn concentrations were significantly higher in participants with TBAD than in healthy controls. Compared with patients with acute myocardial infarction (AMI), the serum concentrations of VEC and Vcn in patients with TBAD were higher, and the Vcn showed significant difference, with statistical significance. Receiver operating characteristic analysis generated areas under the curves for VEC and Vcn that were diagnostic for TBAD (0.599 and 0.655, respectively). The optimal cut-off values were 3.975 ng/μL and 128.1 pg/mL, the sensitivities were 43.0% and 35.0%, and the specificities were 73.3% and 90.0%, respectively. In addition, the use of a combination of serum VEC and Vcn increased the AUC to 0.661, with a sensitivity of 33.0% and a specificity of 93.33%. A high serum Vcn concentration was associated with a higher risk of visceral malperfusion in participants with TBAD (odds ratio (OR) = 1.007, 95% confidence interval [CI]: 1.001–1.013, *p* = 0.014). In participants with refractory pain, the adjusted OR for the serum VEC concentration increased to 1.172 (95% CI: 1.010–1.361; *p* = 0.036), compared with participants without refractory pain. Conclusion: This study is the first to show the diagnostic value of serum VEC and Vcn for AAD and their relationships with the clinical characteristics of patients with TBAD. Thus, VEC and Vcn are potential serum markers of TBAD.

## 1. Introduction

Acute aortic dissection (AAD) is defined as a disruption of the medial layer of the aorta, which is provoked by intramural bleeding, followed by the separation of the aortic wall layers, and the subsequent formation of a true lumen and a false lumen (FL), with or without communication [1]. In most cases, the aortic dissection is initially caused by a tear in the intima, which is followed by the passage of blood into the vascular mesosphere. AADs are commonly divided into type A aortic dissection (TAAD) and type B aortic dissection (TBAD), according to the Stanford classification [2].

As we all know, computed tomography angiography (CTA) is the gold standard for diagnosing aortic dissection. However, in community hospitals or clinics in China where there is no advanced imaging method, biochemical testing may be the key to help medical professionals make a preliminary diagnosis decision whether to send a patient home or refer them to a tertiary center. Any center needs a certain basis for CT examination, and serological screening is an important basis. Compared with CTA, the popularization of serological screening is easier. At the same time, serological examination will be safer and reduce iatrogenic damage for patients who cannot cooperate with a computed tomography angiography (CTA) examination or have clear CTA contraindications (such as reduced creatinine clearance, advanced age, diabetic patients taking metformin, blood pressure higher than 150 mmHg, fasting time less than 4 h, etc.). Several biomarkers have been shown to be clinically useful for the discrimination of TBAD from conditions with similar presentations, such as C-reactive protein (CRP), matrix metalloproteinases (MMPs), soluble elastin fragments (sELAF), D-dimer, smooth muscle myosin heavy chain, calponin, N-terminal pro-brain natriuretic peptide (NT-proBNP), big endothelin-1 (Big ET-1), genetic markers, and so on [3].

An increasing number of studies have indicated that the progression of AAD involves a complex pathogenesis, including inflammation, injury to vascular endothelial and smooth muscle cells, and extracellular matrix degradation. It has been widely reported that endothelial integrity plays a crucial role in the maintenance of normal fluid homeostasis and the capacity of the vessel wall to resist thrombosis and inflammatory reactions [4]. Vascular endothelial cadherin (VEC) is a canonical endothelial-specific cell–cell adhesion protein that is critical for endothelial barrier function and the prevention of vascular inflammation. Multiple regulatory and signaling mechanisms converge on VEC and thereby regulate endothelial barrier function and angiogenic remodeling [5]. Vinculin (Vcn) is a ubiquitously expressed cytoskeletal protein that links transmembrane receptors to actin filaments, and plays a key role in the regulation of cell adhesion, motility, and force transmission [6]. Previous studies have shown that the loss of Vcn contributes to the severe weakening of the extracellular matrix and cell–cell adhesion, which ultimately promotes cancer proliferation and migration [7,8]. Both VEC and Vcn participate in the establishment of the actin cytoskeletal complex, which plays roles in both vascular inflammation and remodeling [9,10,11]. However, to our knowledge, there is a lack of evidence concerning the relationship between AAD and these two endothelial proteins.

In the present study, we measured the serum VEC and Vcn concentrations in patients with TBAD and matched controls and analyzed the clinical relationships between TBAD and these two proteins. We also compared the diagnostic value of serum VEC, serum Vcn, and their combination. Furthermore, we analyzed the relationships between the two proteins and the acute complications of TBAD. These findings provide new insight into both the pathogenesis and clinical characteristics of TBAD.

## 2. Materials and Methods

### 2.1. Study Population

We enrolled 100 patients with TBAD from the First Affiliated Hospital of China Medical University between March 2017 and July 2019. Patients with TBAD were initially admitted to the Emergency Department for evaluation, diagnosed using CTA within 24 h of the onset of symptoms, and classified according to the Stanford standard [12]. The exclusion criteria were chronic aortic dissection, malignancy, autoimmune disorders, severe vascular stenosis, hematological disorders, infectious diseases, coronary heart disorders, severe organ failure, congenital heart disorders, previous aortic surgery, Marfan syndrome, Ehlers–Danlos syndrome, other connective tissue or vascular disorders, and the use of non-steroidal anti-inflammatory drugs or steroids. The demographic and clinical characteristics, risk factors, and laboratory test results for each participant were obtained from their electronic medical records. Laboratory testing was performed according to the hospital laboratory uniform measurement standards, as described previously.

Volunteers who were healthy after a physical examination were enrolled in the control group if a diagnosis of AAD was excluded using imaging examination, including CT or magnetic resonance imaging, or both, at admission. Another control group encompassed AMI patients in the emergency department. (Patients were diagnosed with AMI if they had chest pain lasting > 20 min, diagnostic serial ECG changes comprising new pathological Q waves or ST-segment and T-wave changes, and a plasma creatine kinase-MB elevation greater than twice the normal level or cardiac troponin I (cTnI) level greater than 0.1 ng/mL). They also passed CTA examination to exclude AAD. Blood samples were obtained from 60 healthy volunteers and 30 patients with AMI. The exclusion criteria for the control group were malignancy, infection, a history of the use of medication, and immune-related disorders. Ultimately, 90 control participants were included. The demographic and clinical characteristics of these participants were also collected.

All the participants included in the study gave their written informed consent and the study was approved by the Ethics Committee of the First Hospital of China Medical University (Shenyang, China).

### 2.2. Sample Collection and the Determination of Serum VEC and Vcn Concentrations

The participants underwent venipuncture on an empty stomach on the first morning within 24 h of admission, and the blood samples were collected into EDTA-coated plastic tubes (BD Vacutainer, 5.0 mL). The samples were centrifuged to collect plasma (3000 r/min), which was stored at −80 °C for up to 1 year. The serum concentrations of VEC and Vcn were measured using ELISA kits (VEC, R&D systems, Minneapolis, MN, USA; Vcn, Cusabio, Wuhan, China), according to the manufacturers’ protocols.

### 2.3. Statistical Analysis

Statistical analyses were performed using SPSS 22.0 (IBM Inc., Armonk, NY, USA). Categorical variables are shown as numbers with percentages, and biochemical and clinical data for the two groups were compared using the chi-square test. Continuous variables are shown as mean values with standard deviations. The relationships between continuous variables were analyzed using Spearman’s correlation analysis. Multiple logistic regression models were constructed to assess the relationships of the serum VEC and Vcn concentrations with the risk of TBAD, after adjustment for potential confounding factors. Receiver operating characteristic (ROC) curves and the associated areas under the curves (AUCs), based on logistic models, were used to determine the most appropriate cut-off values and assess the diagnostic performance of serum VEC, Vcn, white blood cells (WBCs), and a combination for TBAD. *p* ≤ 0.05 (two-sided) was considered to represent statistical significance.

## 3. Results

### 3.1. Baseline Clinical Characteristics of the Participants

The detailed demographic characteristics and clinical features of the participants with TBAD and controls are listed in Table 1. No significant differences were observed in age, sex, body mass index (BMI), the prevalence of diabetes mellitus, and the prevalence of smoking between the controls and participants with TBAD (*p* = 0.260, *p* = 0.633, *p* = 0.287, *p* = 0.906, and *p* = 0.290, respectively). Compared with the control group, the participants who had been diagnosed with TBAD had a significantly higher heart rate, white blood cell (WBC) count, and prevalence of hypertension (*p* < 0.001, *p* < 0.001, and *p* = 0.001, respectively) and a lower hemoglobin (Hb) concentration. Compared with the AMI control group, there was no significant difference in these indexes. In the TBAD group, 44 participants experienced acute complications (44%), including refractory pain (34 participants), uncontrollable hypertension despite adequate medical treatment (22 participants), and poor perfusion of the limbs (12 participants) or viscera (26 participants). Refractory pain and uncontrollable hypertension refer to hypertension and pain that cannot be adequately treated by all existing modern pharmacological methods. The occlusion of celiac trunk, superior mesenteric, inferior mesenteric, and/or renal arteries results in severe abdominal pain and decreased urine output, leading to metabolic shock later on. When the flow in the distal abdominal aorta or iliac arteries is compromised, patients may complain of painful, pulseless, or even plegic and cold lower extremities. All participants with TBAD received appropriate medication after the onset of symptoms to control pain, heart rate, and blood pressure to the normal range. In addition, 77 participants subsequently underwent thoracic endovascular aortic repair (TEVAR).

### 3.2. Serum Vcn and VEC Concentrations in TBAD Patients

**Figure 1:**
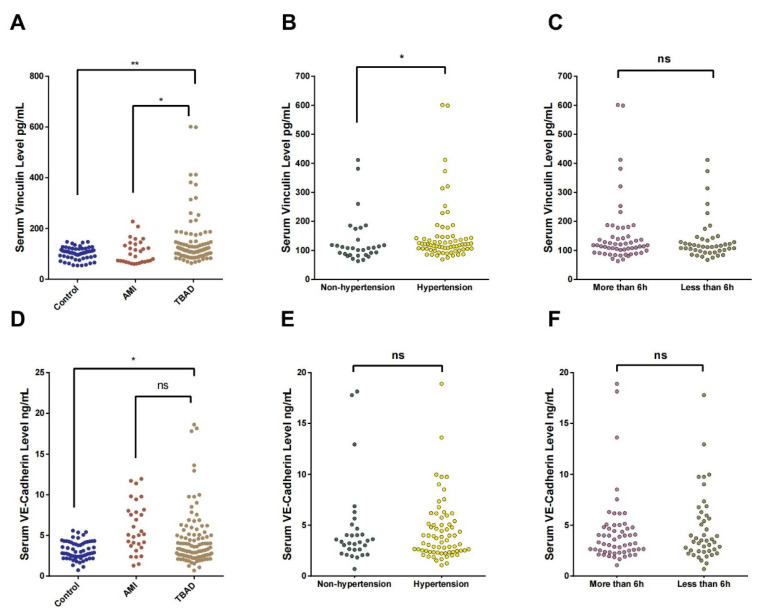
Analysis of serum Vcn and VEC concentrations in different groups. The horizontal axis represents serum Vcn concentrations in mg/L and the vertical axis represents (**A**) control, AMI, TBAD; (**B**) non-hypertension, hypertension; and (**C**) more than 6 h, less than 6 h. The horizontal axis represents serum VEC concentrations in mg/L and the vertical axis represents (**D**) control, AMI, TBAD; (**E**) non-hypertension, hypertension; and (**F**) more than 6 h, less than 6 h. *, *p* < 0.05; **, *p* < 0.01; ns, not significant.

As expected, the serum Vcn concentration was significantly higher in the TBAD group than in the control groups, as shown in Table 1 and Figure 1A (TBAD vs. control: 145.4 ± 9.62 vs. 102.3 ± 3.09, *p* = 0.001; TBAD vs. AMI: 145.4 ± 9.62 vs. 107.32 ± 8.34, *p* = 0.036). In addition, the serum Vcn concentration was significantly higher in participants with a history of hypertension than in those without (151.2 ± 12.26 vs. 132.7 ± 14.81, *p* = 0.043; Figure 1B). However, no significant difference was observed between participants with acute-phase features that persisted for <6 h or >6 h (152.9 ± 14.81 vs. 136.0 ± 11.13, *p* = 0.542; Figure 1C).

The concentration of serum VEC in TBAD group was also significantly higher than that in the healthy control group but not significantly higher than that in AMI control group (TBAD vs. control: 4.49 ± 0.34 vs. 3.15 ± 0.14, *p* = 0.003; TBAD vs. AMI: 4.49 ± 0.34 vs. 5.95 ± 0.56, *p* = 0.763; Table 1 and Figure 1D). However, it did not significantly differ between participants with a history of hypertension and those without (151.2 ± 12.26 vs. 132.7 ± 14.81, *p* = 0.043; Figure 1E) or between participants with acute-phase features that persisted for <6 h or >6 h (152.9 ± 14.81 vs. 136.0 ± 11.13, *p* = 0.542; Figure 1F).

### 3.3. Performances of Serum Vcn, VEC, and a Combination of the Two for the Diagnosis of TBAD

**Figure 2:**
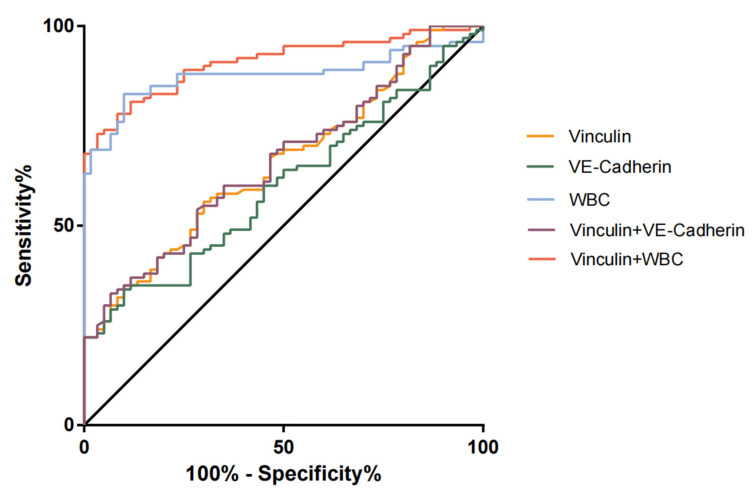
ROC analysis of Vcn, VEC, WBC, and their combination for the evaluation of TBAD. The vertical axis represents the sensitivity, and the horizontal axis represents the specificity.

The ROC analyses of the diagnostic performances of Vcn, VEC, WBC, and their combination for TBAD are shown in Table 2 and Figure 2. The AUCs for Vcn and VEC alone were 0.655 and 0.599, with optimal cut-off values of 128.1 pg/mL and 3.975 ng/μL, respectively. These were associated with sensitivities of 35.0% and 43.0% and specificities of 90.0% and 73.3%, respectively. The AUCs for Vcn and VEC alone were low, but their specificities were quite high. For the combination of Vcn + VEC, the AUC (0.661) was higher than for Vcn or VEC alone, and the specificity of the combination was 93%, which was higher than Vcn or VEC alone. The AUC for WBC was 0.876, with optimal cut-off values of 6.685 × 10^9^/L, associated with sensitivities of 83.0% and specificities of 86.7%. For the combination of Vcn + WBC, the AUC (0.909) was higher than for Vcn or VEC alone, and the specificity of the combination was 96.7%, which was higher than Vcn or VEC alone.

### 3.4. Relationships of Serum Vcn and VEC with Blood Parameters

We next evaluated the relationship between the serum Vcn and VEC concentrations and found a significant correlation between them (r = 0.207, *p* = 0.039). We also found that serum Vcn significantly correlated with neutrophil count, high-density lipoprotein-cholesterol (HDL-C) concentration, and triglyceride (TG) concentration (r = 0.202, 0.235, and −0.320, and *p* = 0.043, *p* = 0.033, and *p* = 0.003, respectively; Table 3). Moreover, there was a significant correlation between the serum concentrations of VEC and D-dimer (r = 0.217, *p* = 0.030; Table 3).

### 3.5. The Relationships of Serum Vcn and VEC with Acute Preoperative Complications of TBAD

We next performed multiple logistic regression analysis to evaluate the relationships of serum Vcn and VEC with the acute preoperative complications of TBAD, with adjustment for age, sex, BMI, smoking, hypertension, and diabetes mellitus (Table 4). A high serum Vcn concentration was associated with a higher risk of poor visceral perfusion (diagnosis by computed tomographic angiography) in participants with TBAD (odds ratio [OR] = 1.007 per unit increase; 95% confidence interval [CI] = 1.001–1.013, *p* = 0.014). In addition, in participants with refractory pain, the adjusted OR for serum VEC concentration increased to 1.172 (95% CI: 1.010–1.361, *p* = 0.036), compared with participants without refractory pain.

## 4. Discussion

To the best of our knowledge, the present study is the first to report that serum Vcn and VEC may represent useful circulating biomarkers of TBAD. TBAD is a serious disease that requires vascular surgery and is characterized by a tear in the descending aorta, high mortality, and morbidity [13]. Endothelial injury is considered to be an important component of the pathogenesis of TBAD [14,15]. Vcn and VEC play crucial roles in the formation and stabilization of epithelial cell–cell adhesion [16]. Therefore, in the present study, the high serum concentrations of Vcn and VEC may reflect the severity of the aortic injury in TBAD.

Vcn is an important component of the focal adhesion complex [9] and exists in active and inactive forms. The active form of Vcn is localized to focal adhesions at membranes and participates in their regulation [17]. The recruitment of Vcn is important for the maintenance of the epithelial barrier, which is achieved by protecting endothelial junctions from opening during force-dependent remodeling [11]. Zemljic-Harpf and colleagues reported that Vcn deficiency contributes to cardiomyopathy [18]. VEC is an endothelial-specific member of the cadherin family that can maintain the stability of endothelial cell–cell junctions [19]. Recent studies have shown that when endothelial junctions are disturbed, some VEC molecules can be released into the blood in a soluble form [20,21]. The loss of VEC induces pathophysiological conditions, including inflammation, vascular leakage, and tumor-associated angiogenesis [22,23]. In the process of tumor metastasis, cancer cells must migrate into blood vessels and lymphatic vessels through extracellular matrix (ECM). This involves the regulation of intercellular adhesion molecules on cell morphology and vascular permeability [7,23]. It has also been previously reported that Vcn can protect VEC junctions from opening during their force-dependent remodeling [16].

It has been reported that mechanical stretch can aggravate AAD in a β-aminopropionitrile-induced rat model [24]. Therefore, increases in the serum concentrations of VEC and Vcn may reflect the dysfunction of endothelial junctions. Furthermore, if there are fewer or weaker Vcn-dependent VEC-based junctions, an appropriate response cannot be mounted to the higher force being exerted on the aortic wall in patients with AAD, which may promote its progression.

Previous studies have shown that when the connections between vascular endothelial cells are damaged, the permeability of endothelium increases, and immune cells are able to penetrate the vascular wall, which reduces its integrity and promotes aortic dissection [25,26,27]. During this process, vascular endothelial inflammation develops, which is characterized by an accumulation of innate immune cells [28].

Serum C-reactive protein (CRP) and D-dimer concentrations are routinely measured to aid in the diagnosis of AAD. However, the serum CRP concentration is rarely high in the acute phase of onset of AD, which implies that it has low diagnostic value for the early diagnosis of AAD [29,30]. In addition, the sensitivity and negative predictive value of serum D-dimer concentration are very high at the time of patient admission, but the associated specificity and positive predictive value are much lower [31,32,33]. Because TBAD affects the aortic wall, biomarkers related to injury of the vascular endothelium may be of clinical value.

In the present study, having identified high serum VEC and Vcn concentrations in most of the participants with TBAD, we next determined the value of serum VEC and Vcn and their combination for the diagnosis of TBAD. According to the ROC curves, both Vcn and VEC have relatively high specificity for the diagnosis of AD, and the specificity of Vcn was higher than that of VEC. However, the sensitivities for the use of both proteins were unsatisfactory (35% and 43%, respectively). Interestingly, the VEC–Vcn combination improved the diagnostic accuracy and sensitivity and yielded a higher AUC (0.661) than VEC or Vcn alone. These findings suggest that serum VEC and Vcn represent non-invasive markers of TBAD, and the use of the two in combination may represent a promising means of improving the diagnosis of TBAD, and especially, the differential diagnosis of a high serum D-dimer concentration. We believe that the development of joint diagnostic kits in the future can further improve diagnostic efficiency. Because first of all, in the primary medical center, the emergency department cannot carry out CTA examination, and even if it is diagnosed, it cannot be treated, so it needs to be referred to the superior medical center. The two indicators in this study can determine whether the patient needs to be referred to the superior medical center. Secondly, for patients with contraindications to CTA (such as taking metformin, blood pressure higher than 150 mmHg, fasting time less than 4 h, etc.), Vcn and VCE can be used for screening to provide diagnostic basis. In addition, considering China’s national conditions, the cost of CTA examination is high, especially in emergency treatment, which cannot be covered by medical insurance. Taking CTA as the preferred examination will increase the economic burden for patients. For AMI, unstable angina, pulmonary tuberculosis and other diseases, there are corresponding diagnostic methods, such as patients’ chief complaint, current medical history, physical examination, myocardial enzyme spectrum, troponin, BNP, ECG, coagulation index, and other routine examination methods. The conclusion of this study supports that VEC and Vcn should be added to the admission examination of patients with chest pain to speed up the progress of diagnosis and treatment and reduce the physical and economic burden on patients.

In the present study, we also found that participants with a history of hypertension had a significantly higher serum Vcn concentration than those without hypertension. The serum VEC and Vcn concentrations correlated significantly, and each concentration correlated with several other blood parameters. VEC concentration significantly correlated with that of D-dimer, and Vcn concentration significantly correlated with neutrophil count and the serum HDL-C and TG concentrations. D-dimer is a cross-linked fibrin degradation product that appears in the serum after thrombolysis, and neutrophil count may reflect the acute-phase inflammatory status in TBAD [34]. In addition, high serum HDL-C and TG concentrations are considered to be the risk factors for cardiovascular disease [35,36,37,38].

Further multiple logistic regression analysis showed that serum VEC and Vcn concentrations were associated with the prevalence of acute preoperative complications in patients with TBAD. Of note, compared with patients with refractory pain, the adjusted OR for VEC concentration in patients without refractory pain increased to 1.172. However, high Vcn concentration is also indicative of a higher risk of poor visceral perfusion in patients with TBAD. Thus, although these parameters do not reflect all the aspects of the condition, it is clear that they provide at least a partial indication of the progress of the disease.

The present study had some limitations. First, the changes in the serum VEC and Vcn concentrations were not assessed. Second, the sample size was relatively small. Third, we were not able to measure many blood parameters in the control group. Fourth, the disease control group in this study only had AMI and did not detect the data of other diseases with chest pain. The differential diagnosis efficiency of the two indexes in this study needs to be further improved. Due to the low sensitivity of VEC and Vcn, normal levels cannot be used to exclude TBAD. In addition, aneurysm is a degenerative process that occurs when smooth muscle cells are lost and the structure of the aortic wall deteriorates, especially in the elastic medium and adventitia. VEC and Vcn play an important role in maintaining the homeostasis of vascular endothelial cell junction, so we infer that they will not be significantly increased in aneurysms and atherosclerosis, although our study did not include aortic aneurysm. However, the future assessment of tissue expression levels and mechanistic research will provide more detailed information regarding the links between serum VEC and Vcn concentration and TBAD.

In conclusion, we have shown that the serum concentrations of both serum Vcn and VEC are significantly higher in patients with TBAD. In addition, there were close associations between Vcn and VEC and preoperative complications. Furthermore, we have provided evidence that both VEC and Vcn represent highly specific biomarkers for TBAD and may be applicable to the differential diagnosis of TBAD.

## Figures and Tables

**Table 1:** Demographic and characteristics of TBAD patients and controls included in this study.

Variables	Control (*n* = 60)	AMI (*n* = 30)	TBAD (*n* = 100)	*p* Value
				AAD vs. Control
				AAD vs. AMI
Age, years	53.75 ± 1.49	57.33 ± 1.75	56.09 ± 1.35	0.417
				0.898
BMI, kg/m^2^	22.00 ± 0.44	22.88 ± 0.63	22.68 ± 0.47	0.432
				0.659
Male, n (%)	48 (80)	24 (80)	83 (83)	0.466
Hypertension, n (%)	25 (41.67)	12 (40)	69 (69)	0.001
Diabetes mellitus, n (%)	8 (13.33)	8 (26.67)	14 (14)	0.204
Smoking, n (%)	19 (31.67)	16 (53.33)	40 (40)	0.138
Heart rate, bmp	82.67 ± 1.29	82.87 ± 2.00	88.17 ± 1.29	0.001
				0.496
WBC, 10^9^/L	5.85 ± 0.10	10.52 ± 0.68	10.05 ± 0.38	<0.001
				0.752
NE, 10^9^/L	3.70 ± 0.09	9.02 ± 0.64	7.99 ± 0.39	<0.001
				0.249
Hb, g/L	151.73 ± 2.27	137.97 ± 3.03	142.13 ± 1.75	0.002
				0.483
Vinculin, pg/mL	102.3 ± 3.09	107.32 ± 8.34	145.4 ± 9.62	0.001
				0.036
VE-Cadherin, ng/uL	3.15 ± 0.14	5.95 ± 0.56	4.49 ± 0.34	0.003
				0.763
CRP, mg/L	-	-	55.23 ± 5.18	-
D-Dimer, ug/mL	-	-	6.26 ± 0.70	-
Management in hospital				
Medical Therapy	-	-	100 (100)	-
TEVAR	-	-	77 (77)	-
Acute phase complications			44 (44)	
Refractory pain	-	-	34 (34)	-
Uncontrollable hypertension	-	-	22 (22)	-
Rapid growing	-	-	15 (15)	-
Limb malperfusion	-	-	12 (12)	-
Visceral malperfusion	-	-	26 (26)	-

Note: BMI, body mass index; WBC, white blood cell; NE, neutrophil; Hb, hemoglobin; CRP, C-reactive protein; TEVAR, thoracic endovascular aortic repair.

**Table 2:** Diagnostic performances of serum vinculin, VE-cadherin, and WBC alone, as well as their combination, for TBAD detection.

Variables	Cut-off Value	AUC (95% CI)	Sensitivity	Specificity	*p* Value
TBAD vs. Ctrl					
Vinculin, pg/mL	128.1	0.655 (0.571–0.739)	35.00%	90.00%	0.001
VE-Cadherin, ng/uL	3.975	0.599 (0.512–0.686)	43.00%	73.30%	0.036
WBC, 10^9^/L	6.685	0.876 (0.820–0.933)	83.00%	86.67%	<0.001
Vinculin + VE-Cadherin	-	0.661 (0.577–0.744)	33.00%	93.33%	<0.001
Vinculin + WBC	-	0.909 (0.865–0.954)	73.00%	96.67%	<0.001
TBAD vs. AMI					
Vinculin, pg/ml	80.57	0.650 (0.522–0.779)	94.00%	46.67%	0.013

**Table 3:** The correlations between vinculin and VE-cadherin levels and laboratory tests in TBAD patients.

Blood Parameters	Vinculin (pg/mL)		VE-Cadherin (ng/μL)	
	R	*p* Value	R	*p* Value
Blood routine				
WBC, ×10^9^/L	0.194	0.054	0.084	0.409
RBC, ×10^12^/L	−0.047	0.649	−0.023	0.826
HGB, g/L	−0.056	0.58	−0.075	0.459
BA, ×10^9^/L	−0.005	0.964	−0.047	0.646
EO, ×10^9^/L	−0.093	0.359	−0.046	0.652
LY, ×10^9^/L	−0.154	0.125	−0.136	0.179
MO, ×10^9^/L	0.090	0.372	−0.059	0.560
NE, ×10^9^/L	0.202	0.043 *	0.111	0.270
MCH, pg	0.058	0.578	−0.048	0.644
MCHC, g/L	−0.156	0.13	−0.008	0.942
MCV, fL	0.121	0.242	−0.055	0.597
HCT, L/L	−0.018	0.861	−0.072	0.489
Blood coagulation function				
APTT, s	−0.176	0.09	−0.046	0.633
PT, s	−0.041	0.693	−0.092	0.376
PTA, %	−0.019	0.855	0.086	0.405
INR	0.018	0.864	−0.065	0.53
D-Dimer, ug/ml	0.048	0.636	0.217	0.030 *
Cardiovascular injury-related parameters				
CK, U/L	−0.007	0.952	0.17	0.162
Inflammatory response				
CRP, mg/L	0.013	0.902	0.065	0.524
Liver function				
ALB, g/L	−0.023	0.828	0.034	0.741
ALP, U/L	−0.008	0.944	−0.106	0.319
ALT, U/L	−0.014	0.895	−0.103	0.321
AST, U/L	0.144	0.19	−0.12	0.277
PA, mg/dL	−0.184	0.128	0.178	0.140
LDH, U/L	0.055	0.646	−0.139	0.242
Serum lipid profile				
HDL-C, mmol/L	0.235	0.033 *	0.033	0.766
TC, mmol/L	−0.083	0.468	0.134	0.244
TG, mmol/L	−0.32	0.003 **	0.069	0.535
LDL-C, mmol/L	−0.137	0.219	0.108	0.336

Note, WBC, white blood cells; RBC, red blood cells; HGB, hemoglobin; BA, basophil; EO, eosinophil; LY, lymphocyte; MO, monocyte; NE, neutrophil; MCH, mean corpuscular hemoglobin; MCHC, mean corpuscular hemoglobin concentration; MCV, mean corpuscular volume; HCT, hematocrit; NLR, neutrophil to lymphocyte ratio; MLR, monocyte to lymphocyte ratio; APTT, activated partial thromboplastin time; PT, prothrombin time; PTA, prothrombin activity, INR, international normalized ratio; CK, creatine kinase; CRP, C-reactive protein; ALB, albumin; ALP, alkaline phosphatase; ALT, alanine aminotransferase; AST, aspartate aminotransferase; PA, pre-albumin; LDH, lactate dehydrogenase; HDL-C, high-density lipoprotein-cholesterol; TC, total cholesterol; TG, triglycerides; LDL-C, low-density lipoprotein-cholesterol. * *p* < 0.05; ** *p* < 0.01.

**Table 4:** The adjusted logistic regression analysis of vinculin and VE-cadherin at baseline and association with TBAD acute-phase complications.

Vinculin	Model 1			Model 2			Model 3		
	β	OR (95% CI)	*p* Value	β	OR (95% CI)	*p* Value	β	OR (95% CI)	*p* Value
Refractory pain	0.003	1.003 (0.999:1.007)	0.175	0.003	1.003 (0.998:1.007)	0.247	0.003	1.003 (0.998:1.007)	0.262
Uncontrollable hypertension	0.005	1.005 (1.001:1.010)	0.024	0.004	1.004 (0.999:1.009)	0.086	0.004	1.004 (0.999:1.010)	0.150
Limb malperfusion	0.004	1.004 (1.000:1.009)	0.075	0.003	1.003 (0.999:1.008)	0.170	0.004	1.004 (0.999:1.010)	0.109
Visceral malperfusion	0.007	1.008 (1.002:1.013)	0.006	0.007	1.007 (1.001:1.013)	0.013	0.007	1.007 (1.001:1.013)	0.014
VE-Cadherin	Model 1		Model 2				Model 3		
	β	OR (95% CI)	*p* Value	β	OR (95% CI)	*p* Value	β	OR (95% CI)	*p* Value
Refractory pain	0.16	1.173 (1.024:1.344)	0.021	0.158	1.172 (1.011:1.358)	0.036	0.159	1.172 (1.010:1.361)	0.036
Uncontrollable hypertension	0.069	1.072 (0.945:1.215)	0.283	0.028	1.029 (0.899:1.177)	0.680	0.027	1.028 (0.892:1.184)	0.706
Limb malperfusion	0.075	1.077 (0.929:1.249)	0.323	0.039	1.040 (0.888:1.218)	0.627	0.044	1.045 (0.889:1.227)	0.595
Visceral malperfusion	0.057	1.059 (0.936:1.197)	0.364	0.026	1.027 (0.902:1.168)	0.690	0.037	1.037 (0.905:1.189)	0.599

β: regression coefficient; CI: confidence interval; Model 1: no adjustments; Model 2: adjusted for age, gender, and BMI; Model 3: additionally adjusted for hypertension, diabetes mellitus, and smoking on the base of Model 2.

## Data Availability

The data used in this study are available from the corresponding author if needed.
